# Suppressive Effect of Juzentaihoto on Vascularization Induced by B16 Melanoma Cells *In Vitro* and *In Vivo*

**DOI:** 10.1155/2012/945714

**Published:** 2011-10-27

**Authors:** Shintaro Ishikawa, Takako Ishikawa, Kazuhito Asano, Hiroshi Fujiwara, Mayumi Okada, Masataka Sunagawa, Tadashi Hisamitsu

**Affiliations:** Department of Physiology, School of Medicine, Showa University, 1-5-8 Hatanodai, Shinagawa-ku, Tokyo 142-8555, Japan

## Abstract

Juzentaihoto (JTT) is well known to be one of Japanese herbal medicines, and used for the supplemental therapy of cancer patients with remarkable success. The present study, therefore, was undertaken to examine the possible therapeutic mechanisms of JTT on cancer using B16 melanoma cell (B16 cell)/experimental mouse system. JTT was well mixed with rodent chow at 3.0% concentrations, and was administered orally ad libitum. Administration of JTT was started one week before tumor cell injection and continued throughout the experiment. Administration of JTT into mice significantly inhibited tumor metastasis in lungs after intravenous injection of 2 × 10^5^ B16 cells in a volume of 50 **μ**L. JTT also significantly suppressed enlargement of tumor size in hind footpad after the subcutaneous injection of 2 × 10^5^ (50 **μ**L) B16 cells. In the second part of experiments, the chamber that containing B16 cells was buried in the murine back. In JTT administrated group, vascular endothelial growth factor (VEGF) of chamber internal fluid significantly decreased, and vascularization of chamber circumference was also inhibited. These results strongly suggest that oral administration of JTT caused decrease in the generation of VEGF, which is responsible for vascularization, and results in inhibition of B16 cell metastasis.

## 1. Introduction

Cancer patients generally undergo surgical therapy, chemotherapy, radiotherapy, or a combination of these treatments. While the effects of these treatments are significant, it is a fact that most patients suffer from side effects, such as high fever, general fatigue, loss of appetite, pancytopenia, and many kinds of infections. 

 In Japan and China, herbal medicines, including Juzentaihoto (JTT), Hochuekkito, and among others, are used as a supplemental therapy for many kinds of chronic diseases such as loss of appetite, anemia, and chilliness of the arms and legs with remarkable success [1, 2]. Recent reports clearly showed that when herbal medicines are used for cancer treatment, many patients experience fewer or diminished side effects induced by western medicine, such as chemotherapy and radiotherapy, and the survival period is longer [3, 4]. It is also reported that herbal medicine can prevent the progression of colon carcinoma, gastric and breast cancer as well as the prevention of these cancer metastasis to the liver, lung, and bone [3, 4]; moreover, hepatocellular carcinoma has been shown to become smaller without severe side effects after the treatment with herbal medicine [5]. Although these reports strongly suggest that herbal medicines will be a good candidate for the treatment of several types of cancer, the mechanisms by which herbal medicines could improve clinical status, including cancer metastasis, of cancer patients.

 Angiogenesis is involved early in tumor progression as well as in sustained growth, invasion of established tumors, and the development of metastasis [6]. The regulation of angiogenesis is closely related to specific angiogenic factors and their receptors [7]. The predominant angiogenic factor is vascular endothelial growth factor (VEGF), a glycoprotein stimulating mitosis of endothelial cells and their migration [7]. Moreover, VEGF increases capillary permeability responsible for cancer metastases [7–9].

 Our previous work clearly showed that oral administration of JTT for 21 days inhibited metastasis of B16 melanoma cells (B16 cell) on lung surfaces through the enhancement of NK-cell activities. However, the influence of JTT on angiogenesis was not clear at present [10].

 In this study, we examined the effects of JTT on the vascularization of B16 cell, as well as its effects on the VEGF production in B16 cells.

## 2. Materials and Methods

### 2.1. Animals

Specific pathogen-free C57BL/6 male mice, 6-week-old, were purchased from Japan CLEA Co., Ltd. (Tokyo, Japan). The animals were maintained at 25 ± 2°C, humidity 50 ± 2%, and a light and dark cycle of 12 hours in our animal facilities. They were randomly divided into groups of 10 mice and fed chow containing 3% JTT [11] or regular diet (control). This study was approved by the Ethics Committee of Showa University for animal experiments (no. 01067).

### 2.2. Preparation of Diet Containing JTT

JTT was provided by Tumura Co. Ltd. (Tokyo, Japan) as a preservative-free pure powder (Table 1). JTT was well-mixed with regular powder diet (CE-2) for maintaining rats and mice (Japan Clea Co. Ltd., Tokyo, Japan) at concentrations of 3.0% [11].

### 2.3. Cell Line (Tumor Cells)

B16 cells, a highly metastatic mice cell line, were purchased from Dai-Nippon Pharmaceutical Co. Ltd. (Osaka, Japan) and maintained with RPMI-1640 medium supplemented with 10% heat-inactivated fetal calf serum (FCS: Flow Laboratories, North Ride, Australia), 100 units/mL penicillin, 100 *μ*g/mL streptomycin, at 37°C in 5% CO_2_ atmosphere.

### 2.4. Assay for Tumor Cell Metastasis

In the first experiments, B16 cells (2 × 10^5^ cells) were injected intravenously into recipient mice in a volume of 50 *μ*L. After 21 days, mice were killed under ether anesthesia and the number of tumor colonies on the lung surface was counted under a dissecting microscope (SZ-60; OLYMPUS Co. Ltd., Tokyo, Japan).

 In the second experiments, B16 cells (2 × 10^5^ cells) were injected subcutaneously into the left hind pad in a volume of 50 *μ*L. After 21 days, the tumor was removed in the knee under a dissecting microscope (OLYMPUS Co. Ltd., Tokyo, Japan). The volume of tumor lumps (cutting departments) was measured by a water displacement method, using a plethysmometer (model no. TK-101P; Muromachi kikai, Tokyo, Japan) [12]. These mice were then maintained for further 21 days and the black dots, showing tumor colony formation on the lung surface, were counted under a dissecting microscope (OLYMPUS Co. Ltd.). In these two experiments, mice were given food-containing JTT and tap water *ad libitum* for two or three weeks starting 7 days before tumor cell injection.

### 2.5. Assay for Cytotoxic Activity of JTT

B16 cells (5 × 10^5^ cells) were introduced into each well of 24 well culture plates that contained 10% and 25% serum prepared from mice treated with JTT for 21 days in triplicate. After 24 hours, the numbers of viable cells were counted with a Countess Automated Cell Counter (Invitrogen Co., Tokyo, Japan) in the presence of trypan blue.

### 2.6. Millipore Chamber (MPC) Implantation

Millipore Chambers (MPCs) [13, 14] containing B16 cells (1 × 10^6^ cells) were implanted into the different experimental groups. The components of a MPC consisted of a plastic holding chamber (Millpore UK Ltd., Watford, UK), two fixation discs, and two 0.45 *μ*m pore size Millipore Filters of 14 mm diameter (Millipore UK Ltd.). B16 cells cultured were placed in the holding chambers which were then closed by sealing a Millipore Filter at both ends using the fixation discs. Recipient mice were anaesthetized using an intraperitoneal sodium pentobarbital. A dorsal longitudinal incision was made and a subcutaneous plane developed into which was inserted a chamber containing B16 cells, and the incision closed with a subcutaneous suture. 

### 2.7. Assay for VEGF Production from B16 Cells

VEGF level in culture supernatants and the MPCs internal fluid was examined using commercially available mouse VEGF enzyme-linked immunosorbent assay (ELISA) kits (R&D Systems, Inc., Minneapolis, Minn, USA) according to the manufacturer's recommendations. The sensitivity of VEGF assay kit was 3 pg/mL.

### 2.8. Determination of the Effect of JTT on *In Vivo* Angiogenesis

Angiogenesis was induced by implantation of the MPCs containing B16 cells (1 × 10^6^ cells/animal) hypodermically on the shaven dorsum skin mouse [15]. After 21 days of MPCs implantation, all animals were sacrificed and B16 cells and body fluid in the chamber were collected. At the same time, the skin adhering to the chamber and blood were collected. The body fluid and serum were used for the estimation of VEGF using ELISA kits according to the manufacturer's recommendation. Dorsum skins removed were washed with PBS and the length of tumor-directed blood vessels per cm^2^ around the tumor was measured using a dissecting microscope (OLYMPUS Co. Ltd.) [16]. The images of mouse hypodermis were magnified to 250 diameters, and the blood vessels length using Digital Scale (FS-DSC101; Firestar Co. Ltd., Hiroshima, Japan) were measured. In addition, these skins were dipped in 4% formalin and used for immunohistological staining [14]. 

### 2.9. Immunohistochemistry for Angiogenesis

The experimental and control skins were stained with the rat primary antibody for mouse CD31 (PECAM-1; BD Biosciences, Tokyo, Japan) to examine the presence of the neogenesis blood vessels in the hypodermis [17, 18]. The shin tissues were washed several times with saline to remove unwanted materials (e.g., blood and connective tissues, etc.), fixed in 4% paraformaldehyde-PBS, followed by 5%, 15%, and 30% sucrose-PBS; specimens were then embedded in Tissue-Tek (Sakura Finetechnical Co. Ltd., Tokyo, Japan) and cut into 10 *μ*m sections. These sections were treated with 3% hydrogen peroxide in PBS to eliminate endogenous peroxidase activity and treated with blocking buffer (PBS containing 5% normal rat serum) for 30 min. Sections were then incubated with CD31 at 4°C for 12 hours. After washing with PBS, the sections were further incubated with secondary antibodies (Histofine Simple Stain Mouse MAX-PO (Rat) kit; Nichirei, Tokyo, Japan) for 30 min. After three rinses with PBS, the sections were incubated with diaminobenzidine substrate (Research Genetics, Huntsville, Ala, USA) for 1 to 2 min. The sections were rinsed with distilled water and counterstained with methyl green. After processing of dehydration with ethanol, the samples were observed under a microscope (BM-2; OLYMPUS Co. Ltd., Tokyo, Japan).

### 2.10. Preparation of JTT-Pulsed Serum and Cell Culture

The mice were given diet containing JTT and tap water ad libitum for 21 days. Blood samples were obtained by cardiac puncture and separated the serum. B16 cells (2 × 10^4^ cells) were grown in 100 *μ*L of PRMI-1640 medium supplemented with 10% heat-inactivated mice serum in a 96-well plate in triplicate. After 24 hours, B16 cells and supernatant were collected and used for the measurement of mRNA expression for VEGF by real-time PCR in triplicate and protein of VEGF by ELISA in triplicate.

### 2.11. PCR Primers and Reagent Kits

The reagents used for mRNA isolation (TaqMan Gene Expression Cells-to-Ct) and real-time RT-PCR (TaqMan Gene Expression Assays) were purchased from Applied Biosystems (Foster City, Calif, USA). These assays were performed according to the manufacturer's instructions [19]. For real-time RT-PCR comparison of gene expression, we selected VEGF gene (vascular endothelial growth factor A: TaqMan Gene Expression Assays; Assay ID: Mm01281449_m1) related to vascularization. GAPDH, glyceraldehydes-3-phosphate dehydrogenase, was used as a housekeeping gene to normalize for RNA loading.

### 2.12. mRNA Isolation: Quantitative RT-PCR

Total RNA was isolated from B16 cells using 50 *μ*L Lysis Solution (P/N4383583). Each sample of total RNA was subjected to reverse transcription (RT) using 20 × RT Enzyme Mix (P/N 4383585) and 2 × RT Buffer (P/N43833586) with Applied Biosystems 2720 Thermal Cycler (Applied Biosystems, Foster City,Calif, USA). After the RT reaction, the cDNA templates were amplified by polymerase chain reaction using TaqMan Gene Expression Assays, PCR primers, and RT master Mix (P/N 4369016). Predesigned and validated gene-specific TaqMan Gene Expression Assays [19, 20] from Applied Biosystem were used in duplicate for quantitative RT-PCR according to the manufacturer's protocol. PCR assays were used with the following protocol: 10 min denaturation at 95°C, and 40 cycles of 15 s denaturation at 95°C, 1 min annealing and extension at 60°C. Samples were analyzed using an ABI Prism 7900HT Fast Real-Time PCR System (Applied Biosystems) [20, 21]. Relative quantification (RQ) studies [22] were made from collected data (threshold cycle numbers, referred to as Ct) with ABI Prism 7900HT Sequence-Detection System (SDS) software 2.3 (Applied Biosystem).

### 2.13. Statistical Analysis

Continuous variables were presented as the mean ± standard error of the mean SEM. The statistical significance between the control and the experimental groups was analyzed with analysis of variance followed by Fisher's protested least significant difference test. A *P* value of less than 0.05 was considered statistically significant.

## 3. Results

### 3.1. Suppression of B16 Melanoma Cell Metastasis by JTT

**Figure 1:**
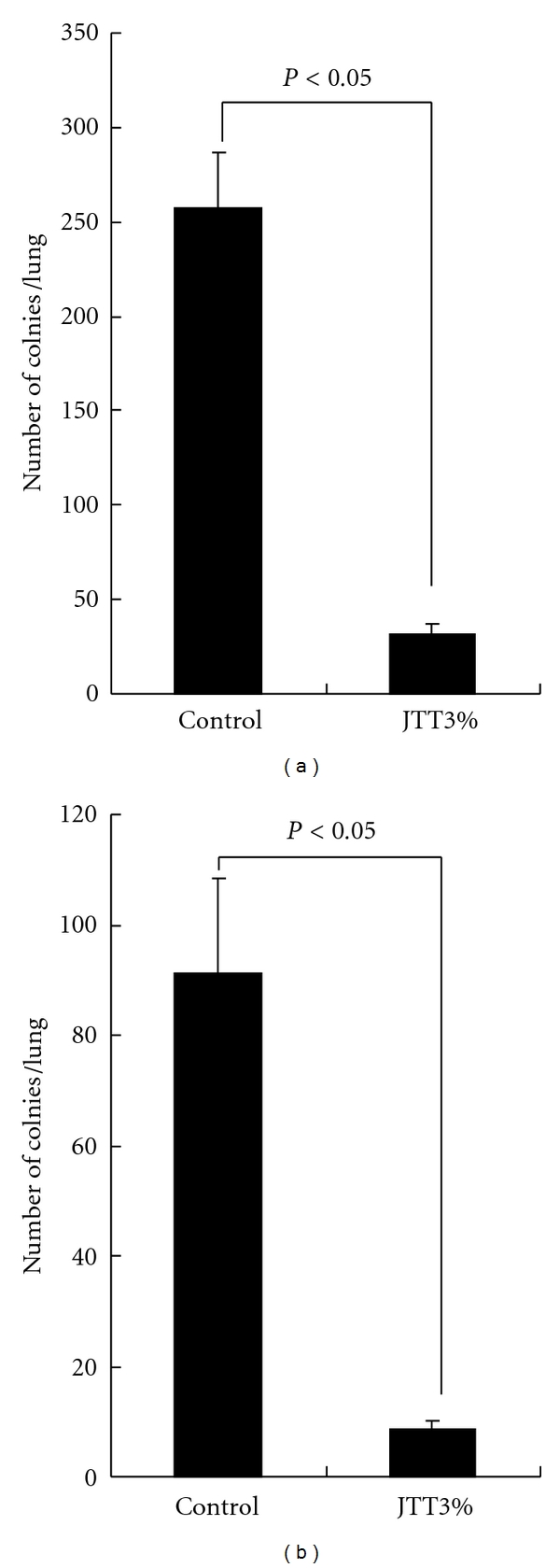
Influence of Juzentaihoto (JTT) on B16 melanoma cell metastasis in mice. C57BL/6 mice were orally administered JTT, which was started one week before injection of 2 × 10^5^ melanoma cells and killed 3 or 6 weeks later to count tumor cell colonies in the lungs. (a) Number of colonies in the lungs 3 weeks after intravenous injection of cells; (b) number of tumor colonies in the lungs 6 weeks after subcutaneous injection of cells. *P < 0.05 versus control (each group: *n* = 10, mean ± SEM).

This experiment was undertaken to examine the influence of oral administration of JTT on tumor cell metastasis using different two types of experimental models. Mice pretreated with 3.0% JTT were injected intravenously with 2 × 10^5^ B16 cells and were killed 21 days later to count the number of tumor cell colonies on the lung surfaces. As shown in Figure 1(a), oral administration of 3.0% JTT could cause significant suppression of B16 cell metastasis. We then examined whether oral administration of JTT could also prevent spontaneous tumor cell metastasis as in the case of intravenous administration of tumor cells. As shown in Figure 1(b), administration of 3.0% JTT into mice could prevent spontaneous B16 cell metastasis from right hind footpad to lung surfaces.

### 3.2. Effect of JTT on Paw Swelling

**Figure 2:**
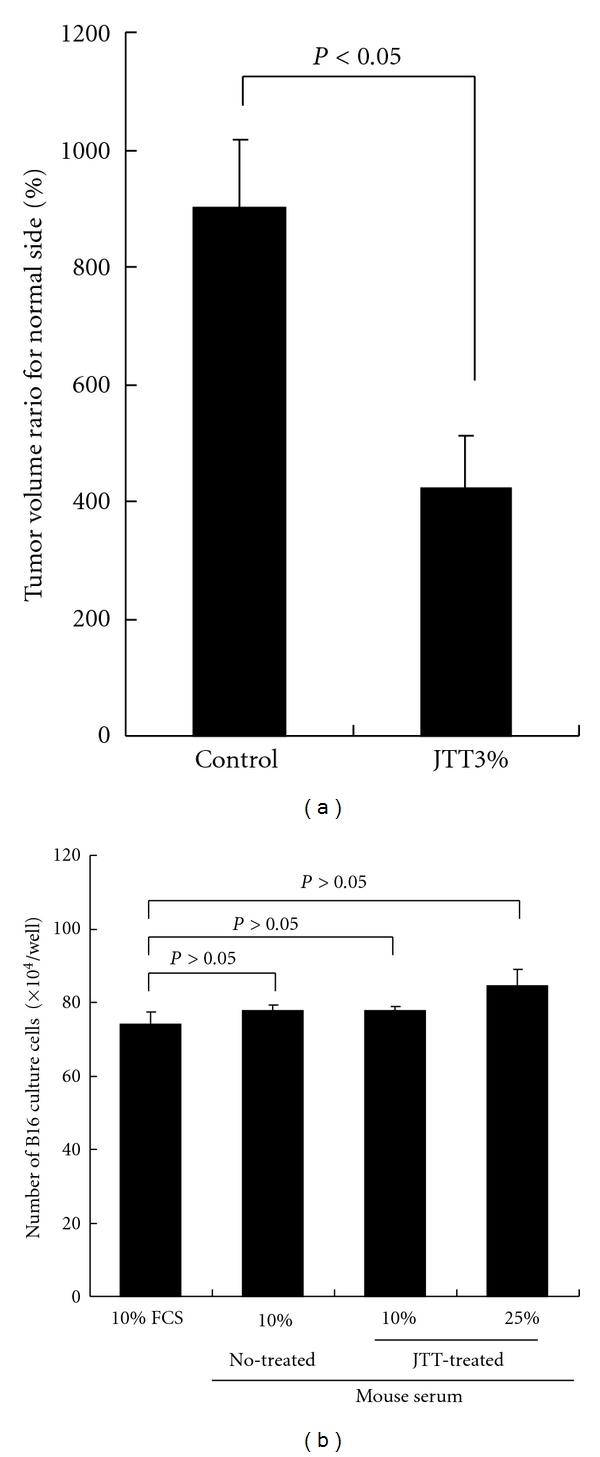
Influence of JTT on tumor cell growth *in vitro* or *in vivo*. JTT administration animal exerts cytotoxic effects on B16 melanoma cells and results in the prevention of tumor cell metastasis. (a) Effect of JTT on paw swelling. *P < 0.05 versus control. (b) Effect of the serum principle of JTT administration animals on B16 cultured cells (each group: *n* = 10, mean ± SEM).

This experiment was undertaken to examine the influence of oral administration of JTT on paw swelling caused by tumor cell growth. Tumor cell growth of mice treated with 3.0% JTT were injected into the palm with 2 × 10^5^ B16 cells and the volume of the palm was measured after 21 days. As shown in Figure 2(a), oral administration of 3.0% JTT could cause significant suppression of paw swelling owing to the tumor growth. 

### 3.3. Effect of Serum Obtained from JTT-Treated Mouse on B16 Cell Viability

The present study was designed to determine whether the serum prepared from mice treated with JTT exerts cytotoxic effects on B16 cells and results in the prevention of tumor cell metastasis. B16 cells at a concentration of 5 × 10^5^ cells/well were cultured in the mouse serum for 24 hours and the numbers of viable cells were counted with trypan blue dye exclusion test. As shown in Figure 2(b), JTT group could not suppress B16 cell growth even when cells were cultured in the presence of 25% mice serum: the number of cells in experimental cultures is almost equal (not significant; *P* > 0.05) to that observed in control cultures.

### 3.4. Effect of JTT on Angiogenesis

**Figure 3:**
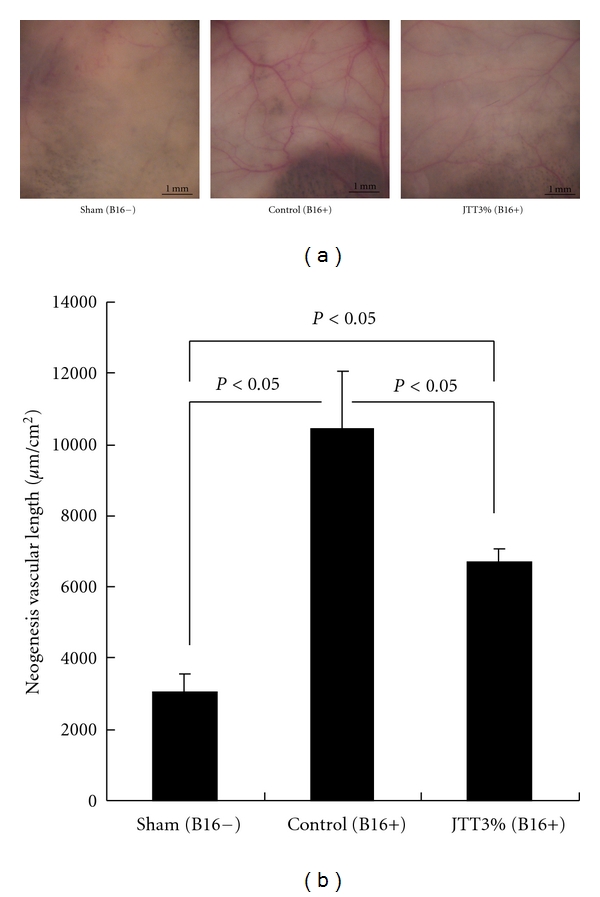
Effect of JTT on angiogenesis (MPCs). The ability of JTT to inhibit *in vivo* tumor-induced angiogenesis was examined by implantation of the MPCs containing B16 cells (1 × 10^6^ cells/animal) hypodermically intradermally on the shaven dorsum of animals. In the sham group, the vascularization occurred mildly. (a) The blood vessels images which were induced by B16-MPCs. (b) Neogenesis vascular length which was induced by B16-MPCs. *P < 0.05 (each group: *n* = 10, mean ± SEM).

The ability of JTT to inhibit *in vivo* tumor-induced angiogenesis was examined by implantation of the MPCs containing B16 cells (1 × 10^6^ cells/animal) hypodermically on the shaven dorsum of animals. In the sham group, the vascularization occurred mildly. In the group that implanted B16-MPCs, the blood vessels, which were induced by the tumor, were significantly reduced in the JTT-treated group (Figure 3(a)). Control animals had the length of 10427.0 ± 1641.7 *μ*m/cm^2^ around the MPCs whereas JTT-treated animals had mere 6711.3 ± 345.4 *μ*m/cm^2^ (Figure 3(b)).

### 3.5. Immunohistochemistry for CD31

**Figure 4:**
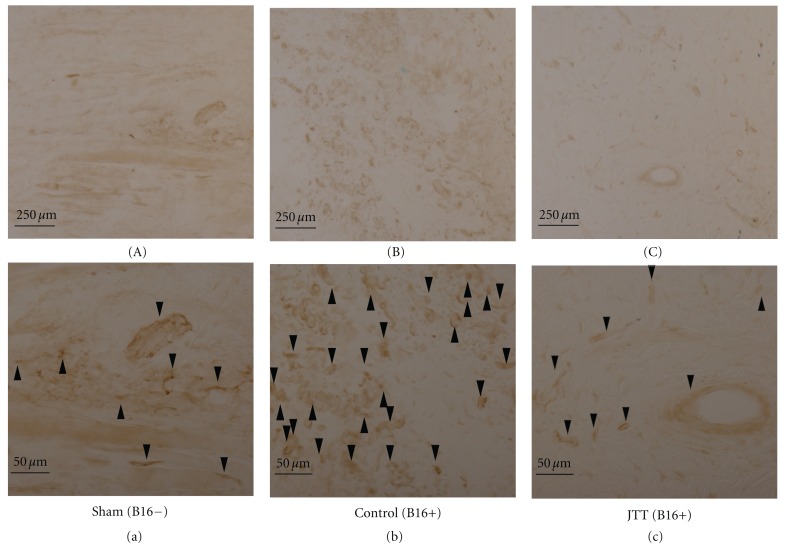
CD31 is expressed on neogenesis blood vessels. Comparison of CD31 expression patterns (brown) with each experimental groups. “a–c” are high magnification of “A–C”. (A–C) Scale bars, 250 *μ*m. (a–c) Arrows are the staining parts of cd31. Scale bars, 50 *μ*m.

Antiangiogenesis is a major anticancer mechanism. Therefore, the skins of MPCs contact hypodermis were evaluated in sections stained with CD31 to further investigate the antiangiogenic effect of JTT. As shown in Figure 4, the (a–c) were high magnification photographs of (A–C), the neogenesis blood vessels (CD31-positive) of the control groups had increased as compared with the sham groups (the CD31-positive cells were shown in arrows). In contrast, vascularization was decreased in the JTT-treated groups.

### 3.6. Influence of JTT on VEGF Production from B16 Cells

**Figure 5:**
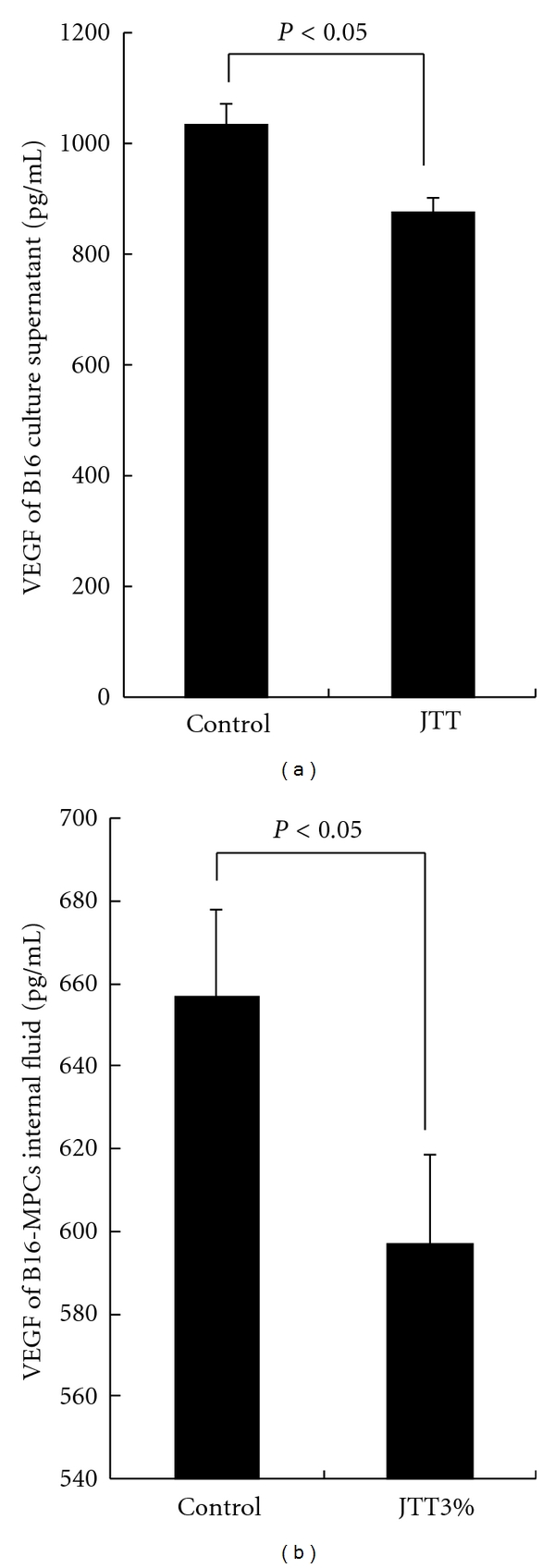
Influence of JTT on VEGF production from B16 cell. The experiment was undertaken to examine the influence of JTT on VEGF production from B16 cells. (a) The VEGF level contents in culture supernatants. (b) The VEGF level contents in the MPCs internal fluid. *P < 0.05 versus control (each sample: *n* = 10, mean ± SEM).

The experiment was undertaken to examine the influence of JTT on VEGF production from B16 cells. The VEGF contents in culture supernatants and the MPCs internal fluid were examined by ELISA. As shown in Figure 5(a), JTT in culture supernatants suppressed the ability of B16 cells to produce VEGF. In addition, as shown in Figure 5(b), JTT treatment in the MPCs internal fluid suppressed the ability of B16 cells to produce VEGF. 

### 3.7. Effect of JTT on the VEGF mRNA Expression

**Figure 6:**
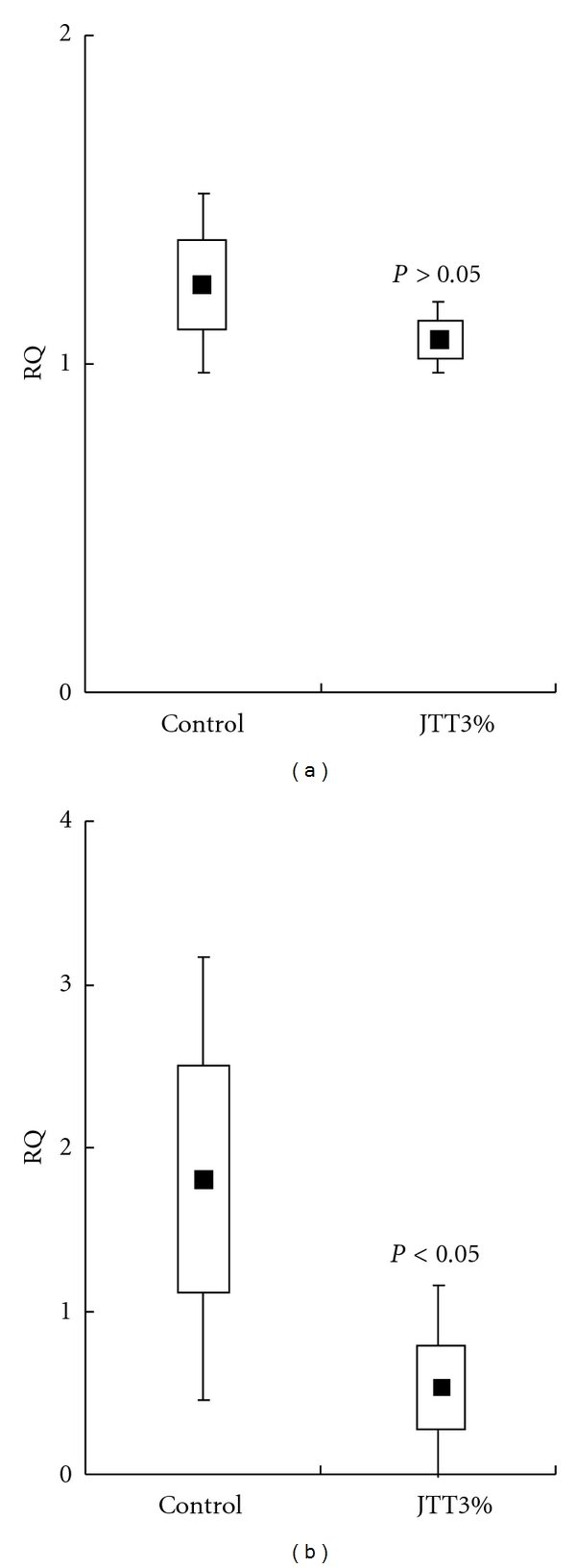
Effect of JTT on the VEGF mRNA expression. The experiment was carried out to examine whether oral administration of JTT could cause decrease in VEGF mRNA expression of the B16 cells. (a) The VEGF mRNA expression of B16 culture cells. (b) The VEGF mRNA expression of the B16 cells contained in the MPCs. *P < 0.05 versus control (each sample: *n* = 10, mean ± SEM).

The final experiment was carried out to examine whether oral administration of JTT could cause decrease in VEGF mRNA expression of the B16 cells. As shown in Figure 6, in the B16 cells contained in the MPCs, the VEGF mRNA expression of the JTT treatment groups had decreased significantly as compared with the control groups.

## 4. Discussion

Herbal medicine is used frequently as a supplemental therapy for many kinds of chronic diseases with remarkable success [1]. In cases of treatment for cancer, herbal medicine is reported to be able to prevent the progression of colon carcinoma, gastric and breast cancer as well as the prevention of the cancer metastasis to the liver, lung, and bone [23]. However, the precise mechanisms by which herbal medicine can alleviate the clinical symptoms of cancer patients such as tumor metastasis, are not well defined. The present study, therefore, was undertaken to examine the possible therapeutic mechanisms of herbal medicine on cancer through the choice of JTT and B16 cell/mouse system *in vivo* and *in vitro*. The present results clearly showed that oral administration of JTT inhibited B16-cell colony formation on the lung surface, when the recipient mice were given tumor cells intravenously (Figure 1(a)). JTT also suppressed spontaneous B16 tumor cell metastasis from hind footpad to the lung surface (Figure 1(b)). The prevention of tumor cell growth and metastasis is well accepted to be through diverse mechanisms, including tumor cell death, apoptosis, and immune-mediated cancer regression. Our results clearly showed the absence of cytotoxic effects of JTT on B16 cells (Figure 2(b)). It is also showed that oral administration of JTT suppressed the paw swelling by B16 cell growth, suggesting that neovascularized mechanisms to promote metastasis are responsible for the prevention of tumor cell colony formation on the lung surface.

In the next study, we focused on the role of JTT during the initial growth phase in the spontaneous metastasis of B16 cells; tumor-induced neovascularization and tumor growth. In the present study, we observed the decrease of neovascularization on tumor tissues treated by JTT administration, especially in the MPCs implanted mice (Figures 3(a) and 3(b)). The experiments of the MPCs implanted B16 cells clearly demonstrate that the high density of CD31-positive vessels in the site of dorsal skin is detectable by immunohistochemical staining. The endothelial cell proliferation (CD31 positive cells) was also inhibited by oral JTT administration. It is important to know about the antiangiogenic effect with JTT administration, because good candidates for antiangiogenic drugs include those available for oral administration for a long term without severe systemic side effects [24]. JTT has satisfied these conditions and could be a candidate for an antiangiogenic material for patients with cancer.

Angiogenesis is composed of several processes: dissociation of pericytes from preexisting vessels, digestion of extracellular matrix with proteases, proliferation, migration and invasion of endothelial cells, and tube formation, and then finally remodeling occurs. VEGF is considered to be secreted from tumor cells in a paracrine fashion to induce blood vessel growth. Even though VEGF is a potent mitogenic stimulation of endothelial cells, several studies have demonstrated the ability of VEGF to function as a survival factor for endothelial cells [25]. Since VEGF is generated from a variety of tumors, it is the most important angiogenic factor associated closely with induction and maintenance of the neovasculature in human tumors [26, 27]. We observed the decrease in VEGF levels in the MPCs contents and culture supernatants treated by JTT (Figure 5). In addition, JTT decreased VEGF mRNA expression in B16 cells (Figure 6) prepared from mice pretreated with JTT for 21 days, but did not culture B16 cells with JTT-treated mouse serum. The reasons for this discrepancy in *in vitro* experiments are not dear at present. The process of protein synthesis requires two different steps. In the first step, so-called transcription, mRNA is synthetized from DNA in the nucleus. mRNA formed then comes out through nuclear membrane into cytoplasm where it attaches to mRNA-binding site on ribosome and starts protein synthesis which is called translocation step. Therefore, there is a possibility that short-term (within 24 h) exposure of B16 cells with metabolized JTT *in vitro* could inhibit only translocation step. Further experiments are needed to clarify this point. These antiangiogenic activities of oral JTT administration may be responsible for the prevention of B16 tumor cell metastasis. 

 We reported that oral administration of JTT into mice caused B16 cell metastasis through the enhancement of IFN-*γ* functions [10]. It is also showed that VEGF-A secretion from corneal fibroblasts induced by proinflammatory cytokine (e.g., TNF-*α* and IL-1) stimulation was suppressed by IFN-*γ* [28]. Taken together, it is suggested that JTT may possess novel pharmacologic properties that interfere with angiogenesis triggered by VEGF secreted from B16 primary oncocytes through the enhancement of IFN-*γ* mechanism.

## Figures and Tables

**Table 1:** Botanical origins of ten crude drugs of JTT from the 2006 Japanese Pharmacopoeia.

Crude drugs	Scientific name of botanical origin	Ratio
*Astragali Radix*	*Astragalus membranaceus Bunge or A. mongholicus Bunge*	3.0
*Cinnamomi Cortex*	* Cinnamomum cassia Blume or other species of the same genus*	3.0
*Rehmanniae Radix*	*Rehamannia glutinosa Liboschitz var. Purpurea Makino or A. glutinosa Liboschitz*	3.0
*Paeoniae Radix*	*Paeonia lactiflora Pallas or allied plants*	3.0
*Cnidii Rhizoma*	*Cnidium officinale Makino*	3.0
*Atractylodis Lanceae Rhizoma*	*Atractylodes lancea De Candolle or A. chinesis Koidzumi*	3.0
*Angelicae Radix*	*Radix Angelica acutiloba Kitagawa or allied plants*	3.0
*Ginseng Radix*	*Panax ginseng C.* *A. Meyer *	3.0
*Hoelen*	*The sclerotium of Poria cocos Wolf*	3.0
*Glycyrrhizae Radix*	*Glycyrrhiza uralensis Fisch, G. grabra Linne, or other species of the same genus*	1.5
